# Supplementary material for: Deletion of wfs1 Impairs Oligodendrocyte Precursor Cells Dorsal Distribution and Myelination Through the wfs1-hmgcs1 Axis in Zebrafish
Source: Biology (Basel). 2026 Aug 16;15(16):1402. doi: 10.3390/biology15161402 (PMC13510151; doi:10.3390/biology15161402)
Supplement: Supplementary file 1 [file biology-15-01402-s001.zip › File S1-Transcriptome sequencing data and differentially expressed genes (DEGs) in wfs1-- zebrafish at 5 dpf/1.GO/wfs1KOvsWT/GSEA/wfs1KO/ESCRT_COMPLEX/ESCRT_COMPLEX(GO_0036452).html]

Details for gene set ESCRT\_COMPLEX(GO:0036452)[GSEA]


|  || Dataset | deg.sample |
| Phenotype | sample.cls |
| Upregulated in class | wfs1KO |
| GeneSet | ESCRT\_COMPLEX(GO:0036452) |
| Enrichment Score (ES) | 0.50831324 |
| Normalized Enrichment Score (NES) | 2.32769 |
| Nominal p-value | 0.0 |
| FDR q-value | 0.011000001 |
| FWER p-Value | 0.011 |
Table: GSEA Results Summary

  

Fig 1: Enrichment plot: ESCRT\_COMPLEX(GO:0036452)
  
Profile of the Running ES Score & Positions of GeneSet Members on the Rank Ordered List

  

| PROBE | DESCRIPTION   (from dataset) | GENE SYMBOL | GENE\_TITLE | RANK IN GENE LIST | RANK METRIC SCORE | RUNNING ES | CORE ENRICHMENT || 1 | 336368 | vps37a |  |  | 138 | 4.569 | 0.2873 | Yes |
| 2 | 336426 | chmp1b |  |  | 758 | 2.459 | 0.4248 | Yes |
| 3 | 100147988 | chmp6a |  |  | 2217 | 1.281 | 0.4608 | Yes |
| 4 | 767667 | mvb12ba |  |  | 2871 | 1.066 | 0.5083 | Yes |
| 5 | 566797 | zgc:114173 |  |  | 6585 | 0.529 | 0.4256 | No |
| 6 | 100004330 | mvb12bb |  |  | 13174 | 0.131 | 0.2272 | No |
| 7 | 558626 | dennd4c |  |  | 13781 | 0.103 | 0.2147 | No |
| 8 | 445397 | chmp6b |  |  | 14023 | 0.091 | 0.2130 | No |
| 9 | 445020 | pdcd7 |  |  | 14286 | 0.079 | 0.2098 | No |
| 10 | 386967 | stam |  |  | 15098 | 0.041 | 0.1870 | No |
| 11 | 777738 | vps37c |  |  | 15374 | 0.029 | 0.1802 | No |
| 12 | 393183 | ubap1 |  |  | 15751 | 0.009 | 0.1689 | No |
| 13 | 393562 | vps28 |  |  | 16239 | -0.009 | 0.1542 | No |
| 14 | 101884856 | si:ch211-284f22.3 |  |  | 18784 | -0.112 | 0.0816 | No |
| 15 | 393535 | chmp1a |  |  | 18849 | -0.116 | 0.0869 | No |
| 16 | 406766 | chmp4bb |  |  | 19021 | -0.122 | 0.0894 | No |
| 17 | 335810 | vps36 |  |  | 19065 | -0.125 | 0.0960 | No |
| 18 | 393164 | chmp4ba |  |  | 19448 | -0.142 | 0.0930 | No |
| 19 | 406621 | chmp3 |  |  | 20867 | -0.208 | 0.0618 | No |
| 20 | 405840 | chmp2bb |  |  | 22045 | -0.270 | 0.0421 | No |
| 21 | 492771 | snf8 |  |  | 23438 | -0.357 | 0.0212 | No |
| 22 | 393754 | chmp7 |  |  | 24474 | -0.418 | 0.0154 | No |
| 23 | 798901 | bc2 |  |  | 25024 | -0.448 | 0.0267 | No |
| 24 | 393456 | mvb12a |  |  | 25258 | -0.464 | 0.0490 | No |
| 25 | 407684 | vps25 |  |  | 28879 | -0.894 | -0.0075 | No |
| 26 | 100004590 | ubap1la |  |  | 30612 | -1.598 | 0.0401 | No |
Table: GSEA details
[plain text format]

  

Fig 2: ESCRT\_COMPLEX(GO:0036452)
  
Blue-Pink O' Gram in the Space of the Analyzed GeneSet

  

Fig 3: ESCRT\_COMPLEX(GO:0036452): Random ES distribution
  
Gene set null distribution of ES for
 **ESCRT\_COMPLEX(GO:0036452)**
